# Response of Rice Nitrogen Physiology to High Nighttime Temperature during Vegetative Stage

**DOI:** 10.1155/2013/649326

**Published:** 2013-08-28

**Authors:** Song Chen, Xiaoguo Zhang, Xia Zhao, Danying Wang, Chunmei Xu, Chenglin Ji, Xiufu Zhang

**Affiliations:** China National Rice Research Institute, Chinese Academy of Agricultural Sciences, Hangzhou, Zhejiang 310006, China

## Abstract

The effects of night temperature on plant morphology and nitrogen accumulation were examined in rice (*Oryza sativa* L.) during vegetative growth. The results showed that the shoot biomass of the plants was greater at 27°C (high nighttime temperature, HNT) than at 22°C (CK). However, the increase in both shoot and root biomasses was not significant under 10 mg N/L. The shoot nitrogen concentrations were 16.1% and 16.7% higher in HNT than in CK under 160 and 40 mg N/L. These results suggest that plant N uptake was enhanced under HNT; however, the positive effect might be limited by the N status of the plants. In addition, leaf area, plant height, root maximum length, root and shoot nitrogen concentrations, soluble leaf protein content, and soluble leaf carbohydrate content were greater in HNT than in CK under 40 and 160 mg N/L, while fresh root volume, root number, and the content of free amino acid in leaf were not significantly different between HNT and CK regardless of nitrogen levels. Moreover, leaf GS activity under HNT was increased at 160 mg N/L compared with that under CK, which might partly explain the positive effect of HNT on soluble protein and carbohydrate content.

## 1. Introduction

High temperature stress is an important yield limiting factor in rice (*Oryza sativa* L.). The global average temperature has been increasing over the past 100 years and is projected to increase in the range of 1.4 to 5.8°C by the end of the twenty-first century at the present rates of greenhouse gas emissions and population growth [1]. In addition, climate models foresee that a relatively greater increase in nighttime temperatures than daytime temperatures will occur because of less radiant heat loss due to increased cloudiness [2]. Over the past century the increases in global daily minimum temperatures were more those twice that of daily maximum temperatures [3]. Evidence of historical yields of rice [4] and wheat [5] shows that cereal yield was strongly correlated with minimum nighttime temperatures rather than daytime maximum temperatures. For example, decreasing rice yields in the Philippines [4] and increasing wheat yields in Mexico [5] were related to increasing nighttime temperatures. 

The influence of high temperatures on carbon and nitrogen metabolism in rice and other crops is well documented. High temperatures damage photosynthetic membranes and cause chlorophyll loss [6], decrease leaf photosynthetic rate, increase embryo abortion [7], lower grain number, and decrease grain filling duration and rates [8–11], resulting in lower grain yield [10, 12–15]. On the other hand, photosynthetic capacity is closely associated with leaf nitrogen [16, 17]. High N level enhances photosynthesis and delays senescence [18]. The two key enzymes involved in assimilating intracellular ammonium into organic compounds are glutamate dehydrogenase (GDH) and glutamine synthetase (GS), which also participate in photosynthesis and carbohydrate metabolism [19, 20]. Heat stress will result in a decrease in leaf N content [18, 21] as well as GDH and GS activities [20, 22] because GDH and GS are associated with amino acid conversion [19] and amino acid composition might be altered due to heat stress, which could promote stress resistance [23]. However, most studies on the effects of high temperatures on crop plants have not differentiated between day- and nighttime temperature regimes. 

Generally, crop development and growth rates and duration of critical phases can be differently sensitive to minimum and maximum temperatures [24]. High nighttime temperatures (HNT) are considered to be disadvantageous because they can stimulate respiration, thereby affecting yield [25]. HNT also affects the leaf photosynthetic rate [26–28], which is attributed to its indirect effects on leaf chlorophyll content [29], leaf area [30], leaf nitrogen concentration (LNC), and various enzymes involved in photosynthesis. Thereby, the effect of HNT on plant growth seems to be closely related with the changes of the coupled plant metabolisms, carbon and nitrogen assimilation. It has been suggested that better understanding of plant responses to HNT is needed to better quantify and reduce uncertainties in climate change impact assessments [24]. However, there has been very little study of the influence of HNT (at constant daytime temperature) on nitrogen and carbon metabolism of rice plants.

The objective of this study was to investigate the combined effects of different nighttime temperatures (22° and 27°C) and different nitrogen levels in hydroponic solution (10, 40, and 160 mg N/L) imposed during the vegetative phase (transplanting to panicle initial stage) on dry matter production, plant nitrogen accumulation, and plant morphological traits under hydroponic solutions. We also investigated the effects of HNT on the content of soluble carbohydrate, soluble starch, soluble protein and free amino acid (FAA) concentration, and the activities of GS, GDH, GPT, and GOT in leaf. The main purpose of this study was to test whether the negative effect of increased nighttime temperature on plant growth could be alleviated by the nitrogen supply in the vegetative stage. 

## 2. Materials and Methods

### 2.1. Plant Material, Growth, and Treatments

Rice (*Oryza sativa* L. cv. N2Y-6) plants were grown hydroponically for 54 days after germination. Seeds were soaked in tap water for 1 day and then germinated at 32°C for 2 days. The germinated seeds were broadcasted on the soil pot and grown in a greenhouse, with a 12 h photoperiod of 1000–1500 *μ*mol quanta m^−2^ s^−1^ of photosynthetic photon flux density (PPFD), 56–85% relative humidity, and an average day/night temperature regime of 33/23°C for 21 days. Seedlings with four leaves were transplanted to a basic nutrient hydroponics solution containing 2.9 mM NH_4_NO_3_, 0.32 mM NaH_2_PO_4_, 1.0 mM K_2_SO_4_, 1.0 mM CaCl_2_, 1.7 mM MgSO_4_·7H_2_O, 9.1 *μ*M MnCl_2_·4H_2_O, 0.52 *μ*M (NH_4_)_6_Mo_7_O_24_·4H_2_O, 18 *μ*M H_3_BO_3_, 0.15 *μ*M ZnSO_4_·7H_2_O, 0.16 *μ*M CuSO_4_·5H_2_O, and 36 *μ*M FeCl_3_·6H_2_O. The pH value of the solution was adjusted to 5.5 using 1 M HCl or NaOH solution as required [31]. Half concentration of the nutrient solution was applied for the first 3 days as pretreatment and then changed to the set nutrient solution. Nutrient solutions were renewed every four days. 

A plastic pot (length × width × height: 40 × 30 × 30 cm) was used, with seedlings planted on the foam and fixed by sponge. The planting spacing was 10 × 10 cm with two seedlings per hill. Three pots with 36 hills were set as one replication. Four environmentally controlled growth chambers (PGB-400; Laifu, China) were used for one rice variety, with two temperature treatments, three nitrogen treatments, and three replications.

**Figure 1:**
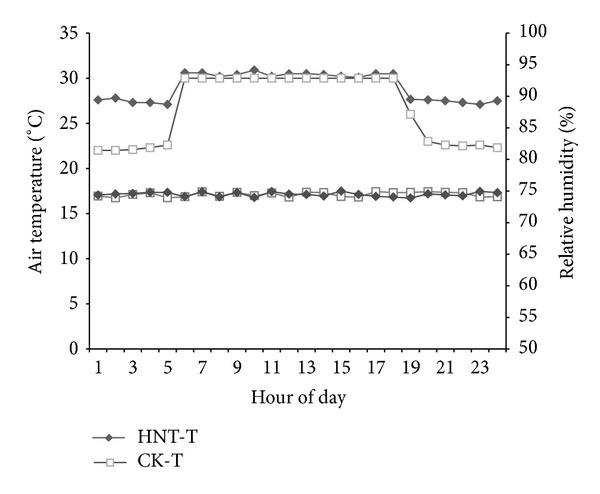
Changes in the air temperature and relative humidity over a day. Plants were grown under two different night temperatures (22 and 27°C) and the same day temperature (30°C).

After the pretreatment, the seedlings were transferred into solutions with different nitrogen concentrations: low (LN, 10 mg N/L), moderate (MN, 40 mmg N/LL), and high (HN, 160 mg N/mL). A total of 18 plots with different nitrogen levels were planted. Two temperature treatments (day/night) were set as HNT (30°C/27°C) and CK (30°C/22°C). Daytime was from 05:00 to 18:00 with a PPFD of 1200 *μ*mol quanta m^−2^ s^−1^ and a relative humidity of 75% throughout the whole day. The details of air/water temperature and relative humidity over a day can be found in Figure 1.

### 2.2. Determination of Plant Biomass, Leaf Area, and Morphological Traits

Twelve plants from each replication were sampled at 54 days after germination. The plants were divided into shoot and root and oven-dried separately at 80°C until the dry weight was consistent. The plant height, maximum root length, and root number were measured manually. Root volume was equal to the volume increase of water in a graduated cylinder when the fresh root was submerged. Leaf area was measured using Licor3100C (LICOR, USA). Then, the dry weight was measured and plant tissues were ground into powder for further analysis. Additional 12 plants were sampled for chemical analysis. Fresh leaf and root were separated immediately and stored at −20°C for further use. 

### 2.3. Nitrogen Concentration, Soluble Protein, Soluble Free Amino Acid, and Soluble Carbohydrate and Starch

Total nitrogen concentration was determined with an autoanalyzer (Foss 2100, FOSS Kjeltec, USA) using the Kjeldahl method following vitriol digestion. Soluble protein content was determined by the protein-dye binding method introduced by Braford [32] using bovine serum albumin as the standard. Amino acid concentration in the leaf was determined by the Ninhydrin method [33] using L-leucine as the standard. Soluble carbohydrates and starch were determined following the method of Zhu et al. [34] using sucrose as the standard. 

### 2.4. GOT, GPT, GS, and GDH Activities

Glutamic-oxaloacetic transaminase (GOT) and glutamic-pyruvic transaminase (GPT) activity were assayed by the method of Wu et al. [35]; one unit (U) of activities was defined as the increase in pyruvic acid content per g protein per hour. Glutamine synthetase (GS) activity was assayed by the method of Spario and Stadtman [36]; one unit (U) of activity was defined as the increase in glutamylhydroxamate per g protein per hour. Glutamate dehydrogenase (GDH) activity was determined following the method of Zhang et al. [37]; one unit (U) of activity was defined as the increase in NADH per g protein per minute. 

### 2.5. SOD, POD, CAT, and MDA

The activity of superoxide dismutase (SOD) was determined according to the method of Dhindsa et al. [38]. One unit of enzyme activity was taken as the activity to cause 50% inhibition. The nitroblue tetrazolium reduction rate was measured by monitoring the absorbance at 560 nm. The activity of peroxidase (POD) was determined in a reaction solution composed of 50 mM PBS (pH 7.0), 2 mM H_2_O_2_, 2.7 mM guaiacol, and 0.05 mL enzyme extract by monitoring the increase in absorbance at 470 nm due to guaiacol oxidation [39]. The activity of catalase (CAT) was assayed in accordance with the method of Jiang and Huang [40]. The malondialdehyde (MDA) content was determined by the method of Heath and Packer [41]. 

### 2.6. Data Analysis

The experiments were laid out in a complete randomized design and were repeated a total of three times. To test the significance of nighttime temperatures effect on growth development and physiology parameters, the data were statistically analyzed using two-way analysis of variance (ANOVA; SAS statistical analysis package version 9.0, SAS Institute, Cary, NC, USA). The means were separated using Tukey's HSD at *P* < 0.05. The standard errors of the mean were also calculated and presented in the graphs as error bars.

## 3. Results

### 3.1. Morphological Characteristics

Four-leaf age seedlings were transplanted and exposed to HNT (27°C) at three nitrogen levels for about 33 days. Obvious symptoms of the nitrogen levels in rice seedling were observed. For example, there was a difference in plant height, and the leaves turned yellow in the low nitrogen level condition. To illustrate the effect of HNT on the morphological characteristics of rice seedlings, we determined the plant height, maximum root length, total number of roots, root volume, leaf area, and shoot and root dry weights (Table 1). 

Generally speaking, all the morphological characteristics improved along with the nitrogen increase except for the maximum root length. The effect of HNT on morphological traits varied within nitrogen levels. Plant height, leaf area, and shoot dry weight were greater in HNT (27°C) than in CK (22°C) under the nitrogen level sof 160 and 40 mg N/L, but little difference was found with nitrogen of 10 mg N/L. The maximum root length in HNT was increased compared with that in CK in the low nitrogen level condition (10 mg N/L), but little difference was found in plants under 40 and 160 mg N/L. In comparison with 22°C, plant height in 27°C was 11.1% and 6.2% higher under 40 and 160 mg N/L, respectively. The root length was 28.7% greater in 22°C than in 27°C at 10 mg N/L. There was little difference in root volume, root number, and root dry weight between HNT and CK.

### 3.2. Nitrogen Concentration and Accumulation in Shoot and Root

**Figure 2:**
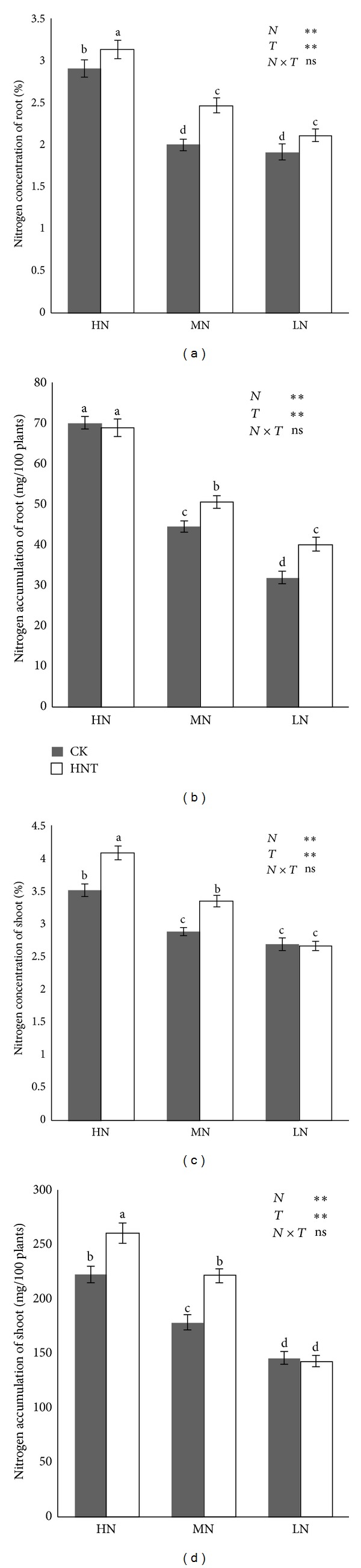
Nitrogen concentration and accumulation in the root and shoot of rice seedling ((a)–(d)). Plants were grown under two temperatures of 22°C (CK) and 27°C (HNT) with three nitrogen levels of 10 mg N/L (low nitrogen level: LN), 40 mg N/L (moderate nitrogen level: MN), and 160 mg N/L (high nitrogen level: HN). Range bars indicate the standard deviation (*n* = 3). ANOVA results are embedded in the figure: ns: not significant; **P < 0.01. Columns with the same letter are not significantly different at *P* < 0.05 by Tukey's HSD.

The HNT effect on nitrogen concentration and accumulation in shoot and root is shown in Figure 2.

Root nitrogen concentrations were 7.72%, 23.5%, and 10.2% greater in HNT than in CK at 160, 40, and 10 mg N/L, respectively. Shoot nitrogen concentrations were 16.1% and 16.7% greater in HNT than in CK at 160 and 40 mg N/L, respectively, but little difference was found at 10 mg N/L. The effect of HNT on nitrogen accumulation and content was similar in root and shoot except that there was no significant difference in root nitrogen accumulation between HNT and CK in the high nitrogen condition (160 mg N/L).

### 3.3. Leaf Soluble Protein, Free Amino Acid, and Soluble Carbohydrate Content

**Figure 3:**
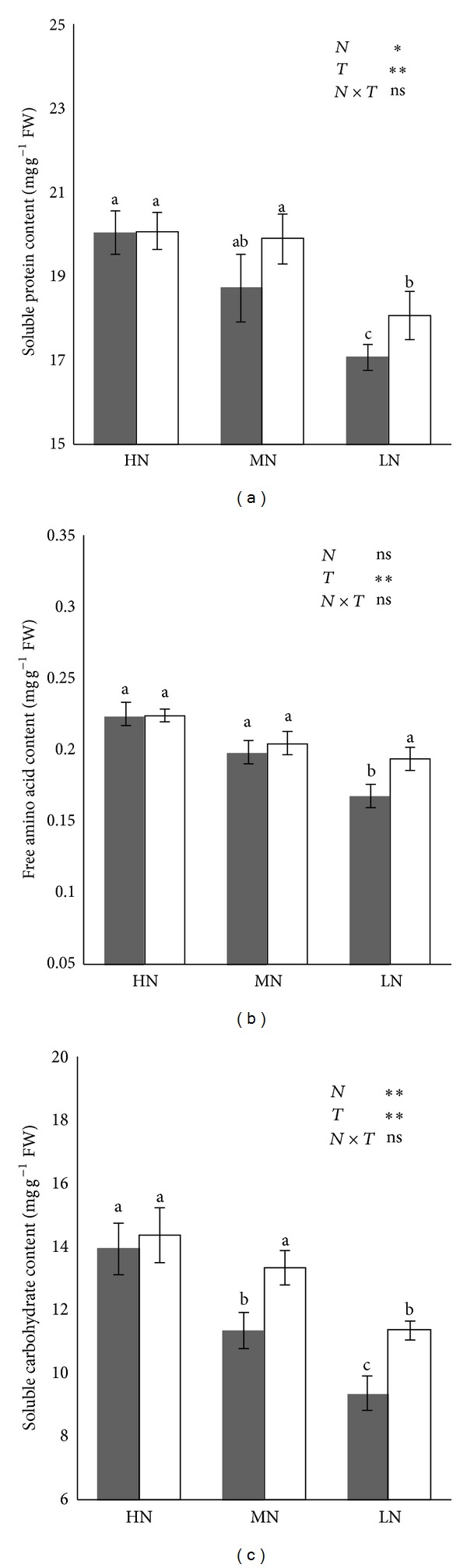
Leaf soluble proteins content (a), soluble sugar content (b), and free amino acid content (c) of rice seedling. Plants were grown under two temperatures of 22°C (CK) and 27°C (HNT) with three nitrogen levels of 10 mg N/L (LN), 40 mg N/L (MN), and 160 mg N/L (HN). Range bars indicate the standard deviation (*n* = 3). ANOVA results are embedded in the figure: ns: not significant; *P < 0.05; **P < 0.01. Columns with the same letter are not significantly different at *P* < 0.05 by Tukey's HSD.

Metabolic productions of leaf nitrogen and carbohydrate metabolism (i.e., soluble protein, free amino acid, and soluble carbohydrates) were determined (Figure 3).

The results showed that there was no significant difference in leaf soluble protein content between CK and HNT at 40 and 160 mg N/L. However, a positive effect of HNT on leaf soluble protein content was significant at 10 mg N/L, being 5.8% greater in HNT than in CK. The effect of HNT on the leaf free amino acid and soluble carbohydrate content varied with N levels. The difference in leaf free amino acid and soluble carbohydrate content between CK and HNT was not significant at 160 mg N/L. A significant difference in soluble carbohydrate content was found at 40 and 10 mg N/L, with HNT being significantly greater than CK. A positive effect of HNT on free amino acid content was only observed at 10 mg N/L.

### 3.4. GOT, GPT, GS, and GDH Activities

**Figure 4:**
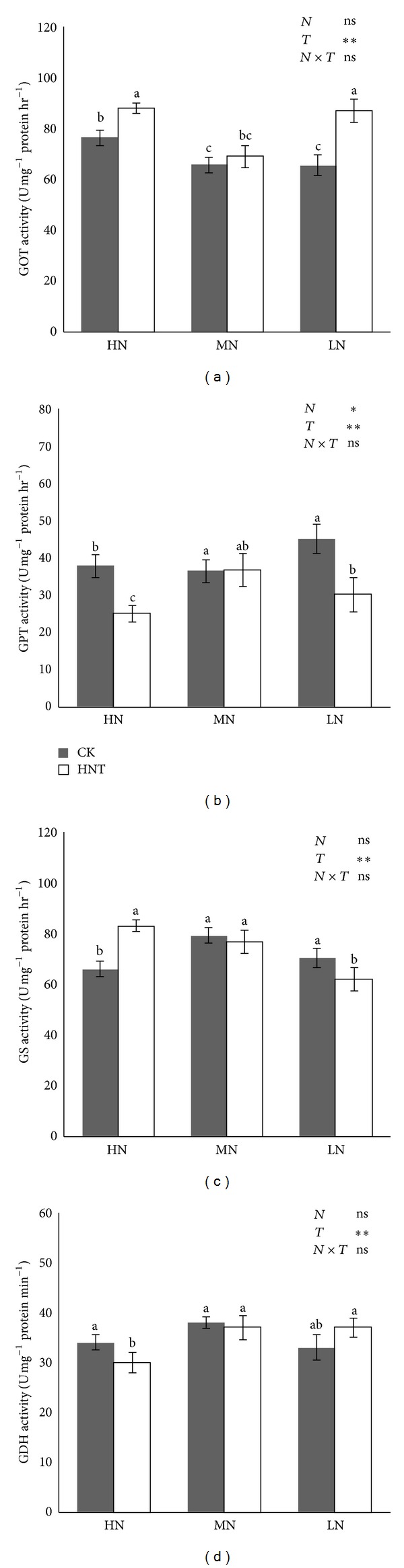
GOT (a), GPT (b), GS (c), and GDH (d) activities of rice seedling leaf. Plants were grown under two different temperatures of 22°C (CK) and 27°C (HNT) and three different nitrogen levels of 10 mg N/mL (LN), 40 mg N/mL (MN), and 160 mg N/mL (HN). Statistical analysis was carried out between temperature treatments using an ANOVA with Tukey's HSD. Columns with the same letter are not significantly different (*P* < 0.05).

Leaf GOT, GPT, GS, and GDH activities were determined to gain a better understanding of the nitrogen assimilation response to HNT (Figure 4). Leaf GOT activity in HNT was increased in the 10 and 160 mg N/L conditions compared with CK, while the opposite results were found for leaf GPT. HNT effects on GS and GDH activities were altered by nitrogen levels. Leaf GS activity under HNT was increased at 160 mg N/L and stayed consistent at 40 mg N/L, but decreased at 10 mg N/L compared with that under CK. The effect of HNT on leaf GDH activity was the opposite at 160 mg N/L but no significant difference was found at 40 and 10 mg N/L between HNT and CK. 

## 4. Discussion

### 4.1. High Nighttime Temperature Effect on Plant Biomass, Leaf, and Root Traits

A high temperature effect on plant biomass has been well documented. Morita et al. [42] found that high temperature accelerates the growth development, leading to decreased accumulation of photosynthates in the sheath, and promotes leaf area, thereby affecting the ultimate grain yield [42]. However, the effect of elevated nighttime temperature on plant biomass was just the opposite. Tanaka et al. [43] observed that biomass production increased with high nighttime water temperatures and that the leaf stage was also promoted. Hussey [44] found that elevated night temperatures in the range of 15–30°C contribute to an increase in the biomass production of tomato plants. Similar effects of night temperature were also found for *Galega officinalis* and *Medicago sativa* in the range of 4–25°C, regardless of the daytime temperature [45]. In the current study, increased biomass under HNT was observed in the shoots of plant exposed to a nitrogen supply of 40 and 160 mg N/L, which was consistent with the previous reports. However, little difference between HNT and CK was found in root biomass (Table 1).

Furthermore, leaf area was also promoted under HNT from 22° to 27°C. The increase in biomass production was attributed to an increase in leaf development. High night temperatures appear to stimulate cell division in the meristems of the leaf and might enlarge thinner leaves at the level of the single leaf [46]. On the other hand, Osone et al. [47] pointed out the importance of leaf-root interactions and found that the N absorption rate of the root was well correlated with leaf development. In this study, the increase in root biomass was not significant at 10 mg N/L, suggesting that N uptake is required to maintain a good correlation between leaf development and RGR under HNT. 

High night temperature may also affect leaf or root traits during the vegetative stage and thus indirectly affect the grain yield [42]. Cutler et al. [48] stated that elevated night air temperature accelerates the leaf elongation of rice. Kanno et al. [49] found greater SLA in HNT plants, and Tsunoda [50] demonstrated that the leaf emergence rate increased when nighttime water temperatures increased. However, root traits were seldom reported. In the current research, we found that root biomass was not significantly different between 22° and 27°C, and similar results were found in fresh root volume and root number, regardless of the nitrogen level. We also found increased plant height, maximum root length, and leaf area in HNT (27°C) compared with CK (22°C) for plants exposed to nitrogen levels of 160 and 40 mg N/L. The maximum root length in HNT was also increased compared with that in CK in the low nitrogen level condition (10 mg N/L), but little difference was found in plants under 40 and 160 mg N/L. These results suggest that the HNT effect on shoot and root development was not consistent during the vegetative stage. 

### 4.2. High Nighttime Temperature on Rice N Metabolism

The effect of HNT on photosynthesis was attributed to its indirect effects on leaf chlorophyll content [29], leaf area [30], leaf nitrogen content (LNC), various enzymes involved in photosynthesis, or a combination of these factors. In this study, root and shoot nitrogen concentration and accumulation were analyzed. The root nitrogen concentrations were 7.72%, 23.5%, and 10.2% greater in HNT than in CK at 160, 40, and 10 mg N/L, respectively, while shoot nitrogen concentrations were 16.1% and 16.7% greater in HNT than in CK at 160 and 40 mg N/L, respectively. Similar results were found for nitrogen accumulation (Figure 2).

These results indicate that plant N uptake was enhanced under elevated nighttime temperature, similar to findings for spring wheat [51]. Leaf nitrogen plays a key role in carbohydrate assimilation and protein synthesis [52]. In the present study, high nighttime temperature increased leaf soluble carbohydrate content at 10 and 40 mg N/L and soluble protein content and free amino acid at 10 mg N/L (Figure 3). However, differences in leaf soluble protein content, free amino acid content, and soluble carbohydrate content between HNT and CK were not significant at 160 mg N/L. It may be that increased accumulation of nitrogen under HNT conditions is related to the N supply level. Increases in the level of proteins or free amino acids were the result of the overall activities of protein synthesis which, in turn, may have an impact on plant nitrogen response or physiological tolerance to high temperatures [53]. 

The key enzymes involved in assimilating intracellular ammonium into organic compounds are glutamine synthetase (GS) and glutamate dehydrogenase (GDH). GS plays an essential role in the metabolism of nitrogen by catalyzing the condensation of glutamate and ammonia to form glutamine [54], while GDH converts glutamate to *α*-ketoglutarate. Both enzymes participate in photosynthesis and carbohydrate metabolism [19, 20]. Although a high N level enhances photosynthesis and delays senescence [18], high temperature results in a decrease in leaf N content [18, 21] and GS and GDH activities [20, 22]. This is important because GDH and GS can be associated with amino acid conversion [19], and amino acid composition might be altered due to heat, which could promote stress resistance [23]. In the present research, the HNT effect on GS and GDH activities was altered by nitrogen levels. Leaf GS activity under HNT increased at 160 mg N/L and remained consistent at 40 mg N/L but decreased at 10 mg N/L compared with that under CK. The effect of HNT on leaf GDH activity was opposite at 160 mg N/L, and no significant difference was found at 40 and 10 mg N/L between HNT and CK. Further studies are needed to investigate the mechanism or mechanisms of the action of GS and GDH and its possible relation to HNT.

**Figure 5:**
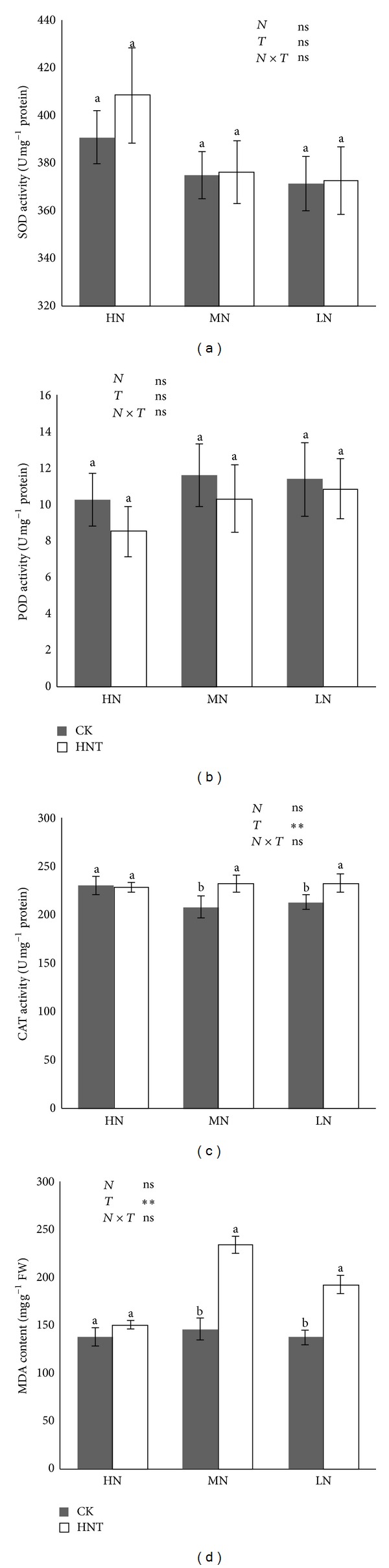
SOD, POD, and CAT activities and MDA ((a)–(d)) content of rice seedling leaf. Plants were grown under two different temperatures of 22°C (CK) and 27°C (HNT) and three different nitrogen levels of 10 mg N/mL (LN), 40 mg N/mL (MN), and 160 mg N/mL (HN). Statistical analysis was carried out between temperature treatments using an ANOVA with Tukey's test. Columns with the same letter are not significantly different (*P* < 0.05).

As an environmental stress, HNT might enhance leaf senescence, involving protein degradation, such as Rubisco [55]. Reactive oxygen species (ROS) production (i.e., superoxide dismutase (SOD), catalase (CAT), peroxide dismutase (POD), and malondialdehyde (MDA)) has the strongest correlation with resistance to oxidative stress. In the present study, no significant difference was found in SOD and POD between CK and HNT regardless of nitrogen levels. However, plants exposed to HNT were significantly greater in MDA and CAT than in CK at 40 and 10 mg N/L (Figure 5). These results indicate that HNT stress on plants was slight, especially when the N supply was sufficient. However, the rice seedlings' defense mechanisms could be stimulated when the N supply is limited, which will, in turn, affect the nitrogen accumulation.

## Figures and Tables

**Table 1:** Night temperature effect on the plant height, maximum root length, root volume, root number, leaf area, and shoot and root dry weights of rice seedlings.

Nitrogen level (N)	Temp. (*T*)	Plant height (cm)	Maximum root length (cm)	Root volume (cm^3 ^plant^−1^)	Root number (plant^−1^)	Leaf area (cm^2^ plant^−1^)	Shoot dry weight (g/100 plants)	Root dry weight (g/100 plants)
160	22°C	42.87 ± 1.49b	15.58 ± 3.10b	0.92 ± 0.08a	92 ± 16a	49.3 ± 8.3b	5.82 ± 0.20b	2.41 ± 0.21a
	27°C	45.53 ± 1.06a	17.38 ± 3.75b	1.01 ± 0.11a	83 ± 14a	55.9 ± 6.8a	6.40 ± 0.28a	2.20 ± 0.19a

40	22°C	39.88 ± 2.43b	17.46 ± 2.61b	0.82 ± 0.14ab	72 ± 20ab	42.8 ± 3.6c	5.51 ± 0.10c	2.23 ± 0.15a
	27°C	44.33 ± 1.82a	20.58 ± 2.56ab	0.80 ± 0.09b	85 ± 18ab	48.7 ± 9.1b	6.01 ± 0.19b	2.05 ± 0.03ab

10	22°C	40.71 ± 1.65b	18.63 ± 2.10b	0.75 ± 0.13b	66 ± 16b	35.3 ± 3.6d	5.02 ± 0.26d	1.97 ± 0.11bc
	27°C	40.17 ± 1.77b	23.99 ± 2.46a	0.72 ± 0.14b	56 ± 12b	39.6 ± 6.8c	5.27 ± 0.10d	1.68 ± 0.01c

ANOVA	N	∗	∗∗	∗∗	∗∗	∗∗	∗∗	∗∗
	*T *	∗∗	∗∗	ns	ns	∗∗	∗∗	∗∗
	N ∗ *T *	ns	ns	ns	ns	ns	ns	ns

Rice seedlings were grown under two different night temperatures of 22°C and 27°C with three nitrogen levels of 10 mg N/L, 40 mg N/L, and 160 mg N/L. Values were expressed as means ± standard error (*n* = 3), and the same small letter in the same column means that the values are not significantly different at *P* < 0.05 by Tukey's HSD.
